# Long-term dental intervention and laboratory examination in a patient with Vitamin D-dependent rickets type I: A case report

**DOI:** 10.1097/MD.0000000000022508

**Published:** 2020-10-09

**Authors:** An-Qi Liu, Li-Shu Zhang, Hao Guo, Mei-Ling Wu, Tian-Yi Li, Kun Xuan, Ke-Wen Wei

**Affiliations:** a State Key Laboratory of Military Stomatology and National Clinical Research Center for Oral Diseases and Shaanxi Clinical Research Center for Oral Diseases, Department of Preventive Dentistry, School of Stomatology; b Department of Dentistry, Hospital of Tangdu, Fourth Military Medical University, Xi’an, Shaanxi Province, China.

**Keywords:** dental intervention, tooth, Vitamin D-dependent rickets type I

## Abstract

**Rationale:**

Vitamin D-dependent rickets type I (VDDR-I) is a rare form of rickets, which is an autosomal recessive disease caused by 1α-hydroxylase enzyme deficiency. However, long-term dental management and microscopic morphology of teeth remain largely unclear.

**Patient concerns:**

We report the case of a 10-year-old Chinese boy complaining of yellowish-brown teeth with extensive caries.

**Diagnoses:**

Clinical and laboratory examinations were performed, and VDDR-I was confirmed. Scanning electron microscopy confirmed amelogenesis imperfecta.

**Interventions:**

The patient had been taking drugs intervention for VDDR-I from the age of 3 years. The decayed teeth were treated, and metal-preformed crowns were placed to prevent further impairment. Sequence tooth extraction and remineralization therapy were also performed.

**Outcomes:**

After 3 years of follow-up, the patient exhibited normal tooth replacement and an acceptable oral hygiene status. However, the new erupted teeth had amelogenesis imperfecta.

**Lessons:**

This case is the first to confirm amelogenesis imperfecta in a patient with VDDR-I that was not prevented by drug intervention. Importantly, it provides evidence that long-term dental intervention in patients with VDDR-I can result in an acceptable oral hygiene status. Therefore, early and long-term dental intervention is necessary in VDDR-I patients.

## 1 Introduction

Vitamin D-dependent rickets type I (VDDR-I) is a rare type of rickets caused by mutation of the *CYP27B1* gene, which leads to 1-α-hydroxylase enzyme deficiency and conversion disorder of 25-OH vitamin D to 1,25-(OH)_2_ vitamin D.^[1]^ Laboratory findings include hypocalcemia, hypophosphatemia, elevated serum parathyroid hormone (PTH), low serum 1,25-(OH)_2_ vitamin D, and normal or increased 25(OH) vitamin D.^[2]^ Clinical manifestations of VDDR-I display the typical characteristics of rickets, including bowleg, growth retardation and ectodermal dysplasia.^[3]^ Although dental abnormalities have been reported in many types of rickets such as vitamin D-dependent rickets type II (VDDR- II),^[4]^ X-linked hypophosphatemia (XLH)^[5]^ and vitamin D–resistant rickets (VDRR),^[6]^ the dental manifestation of VDDR-I and its long-term dental intervention remain largely unclear. Considering that dental abnormalities in children affect chewing ability, pronunciation, facial appearance, and mental health, it is important to provide information on diseases with dental problems for pediatricians and dentists to effectively manage these patients.^[7,8]^

Herein, we report a case of successful dental prevention in a VDDR-I patient with severe oral problems, including amelogenesis imperfecta, tooth decay, and delayed tooth development. Furthermore, scanning electron microscopy (SEM), hematoxylin-eosin (HE) staining and Masson staining were performed to analyze the morphology of the deciduous teeth.

The patient's parent provided informed consent to publish the case. This study was approved by the Institutional Review Board and the Ethics Committee at Fourth Military Medical University.

## 2 Case report

We report the case of 10-year-old Chinese boy who was referred to the Department of Pediatric Dentistry, School of Stomatology, Fourth Military Medical University with complaints of yellowed teeth at the age of seven years. The patient had been diagnosed with VDDR-I at Peking Union Medical College Hospital at 3 years of age with typical osseous and serological changes, such as rachitic rosary, bowleg, low levels of serum calcium, and 1,25-(OH)_2_ vitamin D; as well as high levels of PTH and alkaline phosphatase. After the administration of calcium carbonate and vitamin D3 tablets for 7 years, both laboratory findings and clinical features appeared to be normal (Table 1). According to the patient's mother, there were no similar symptoms present in other family members.

**Table 1:**
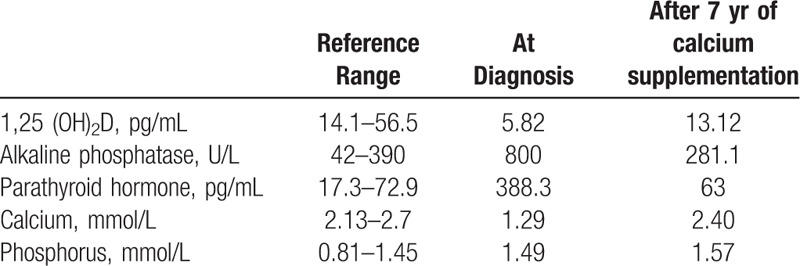
Laboratory studies performed in the patient before and after calcium supplementation.

**Figure 1:**
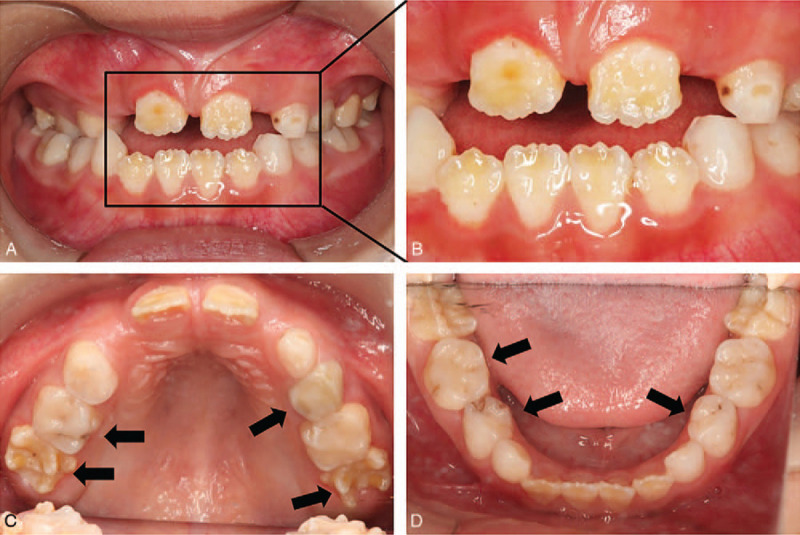
Initial clinical examination. (A) Intraoral views showing yellowish-brown incisors and molars with a large area of enamel exfoliation. (B) Image on the right represents the high magnification of the boxed area. (C, D) Decayed teeth are indicated by arrows.

Oral examination was performed. The patient had a mixed dentition and all erupted teeth exhibited dark yellow coloring with varying degrees of enamel defects, particularly the permanent teeth (Fig. 1A, B). Moreover, multiple cavities existed; in the maxillary first molars (16, 26), the right maxillary second primary molar (55), the left maxillary first primary molar (64), and mandibular primary molars (74, 84, 85) (Fig. 1C, D). A panoramic radiograph revealed delayed development of permanent tooth germs. In addition, all erupted teeth showed enlarged pulp chambers with significantly thin layers of the dentin (Fig. 3A).

**Figure 2:**
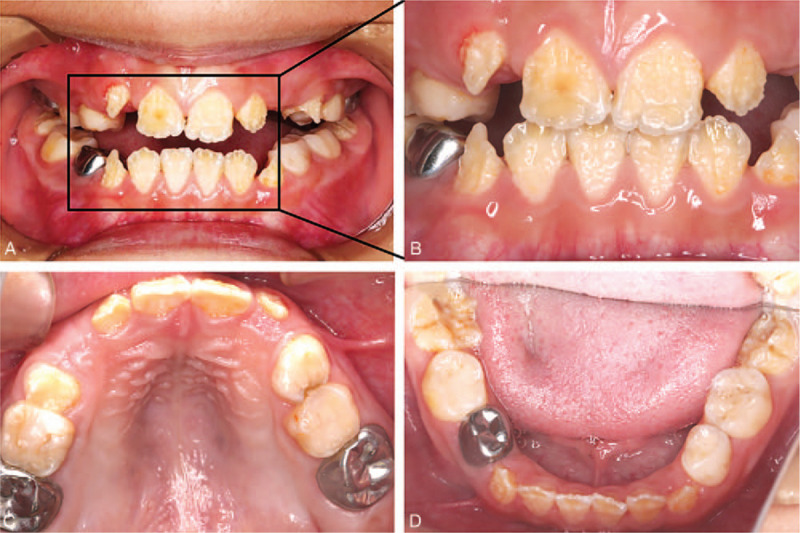
Clinical examination after 3 years of dental intervention. (A) Intraoral views showing newly erupted permanent mandibular canines that are yellowish-brown with enamel defects (B) Image on the right represents the high magnification of the boxed area. (C, D) Acceptable oral hygiene status.

**Figure 3:**
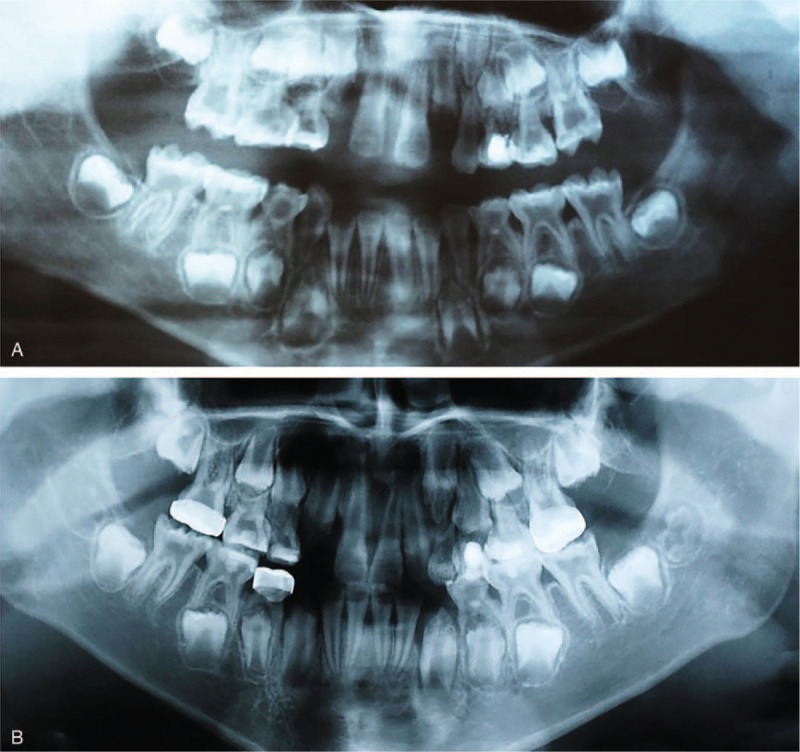
Panoramic radiograph. (A) The panoramic radiograph image of the initial clinical examination. (B) The panoramic radiograph image after 3 years of dental intervention shows the growth of tooth germs.

To treat the oral problems of the patient, we developed a long-term treatment plan. First, we treated the decayed teeth in batches. Primary decayed teeth were treated with routine methods according to the degree of decay. The maxillary first molars (16, 26) exhibited mediated caries. After protecting the pulp with a calcium hydroxide agent and filling the caries with composite resin, we applied metal-preformed crowns to prevent further impairment, as they were crucial in occlusion. Next, remineralization therapy was performed on all the teeth every 6 months as a preventive measure. Furthermore, sequence tooth extraction and oral hygiene guidance were also emphasized throughout our treatment. After 3 years of dental treatment, all permanent teeth exhibited no further damage. The replacement order and eruption time were within the normal range. The metal-preformed crowns remained intact, and there were no signs of secondary caries, pulpitis, or apical periodontitis. However, the new erupted teeth still exhibited amelogenesis imperfecta with yellowish-brown teeth (Figs. 2 and 3B).

**Figure 4:**
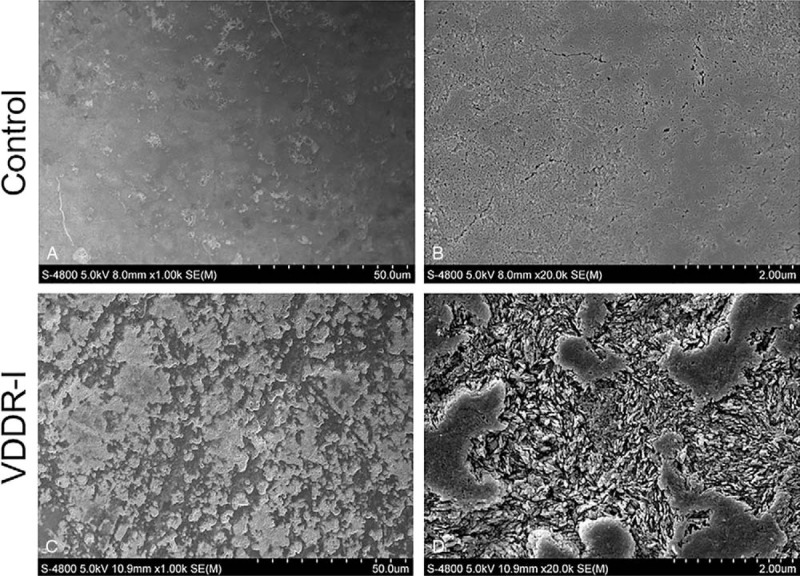
Scanning electron microscopy (SEM) examination of tooth enamel. (A) Control group shows smooth enamel surface. (B) Rough enamel surface observed in the VDDR-I patient. Scale bars: 50 μm. (C, D) High magnification of the enamel surface. Scale bars: 2 μm.

**Figure 5:**
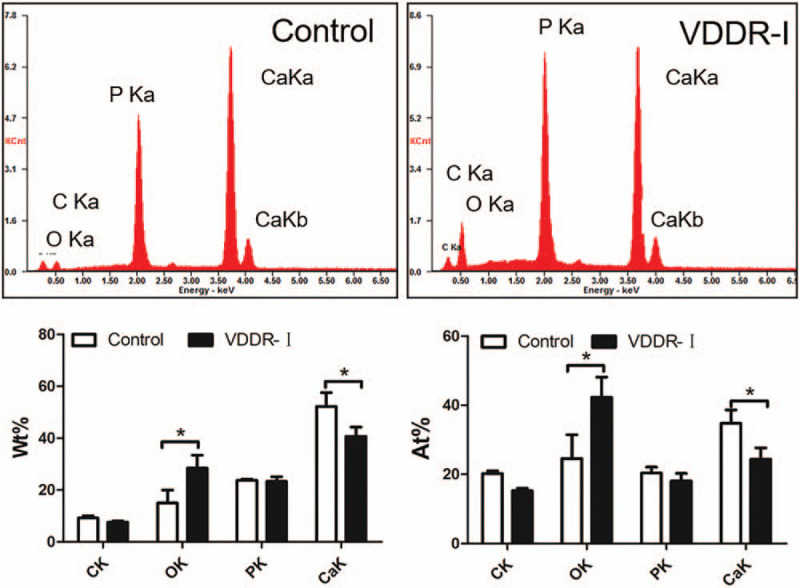
Energy dispersive spectrometer (EDS) analysis showing a reduction in calcium (^∗^, *P* < .05) and elevation of oxygen (^∗^, *P* < .05) in both weight percent (Wt%) and atomic percent (At%) in VDDR-I patient. VDDR-I = vitamin D-dependent rickets type I.

During the treatment, we extracted and collected loose primary canines to facilitate teeth orderly eruption. Additionally, we collected loose primary canine from healthy patients as a control group. SEM was used to evaluate enamel structures. The result confirmed amelogenesis imperfecta, displaying a rough surface and an irregular structure caused by enamel hypoplasty (Fig. 4). Further energy dispersive spectrometry analysis of the enamel showed a decrease in the percentage of calcium (*P* < .05) but an increase in the percentage of oxygen (*P* < .05) in VDDR-I teeth (Fig. 5).

**Figure 6:**
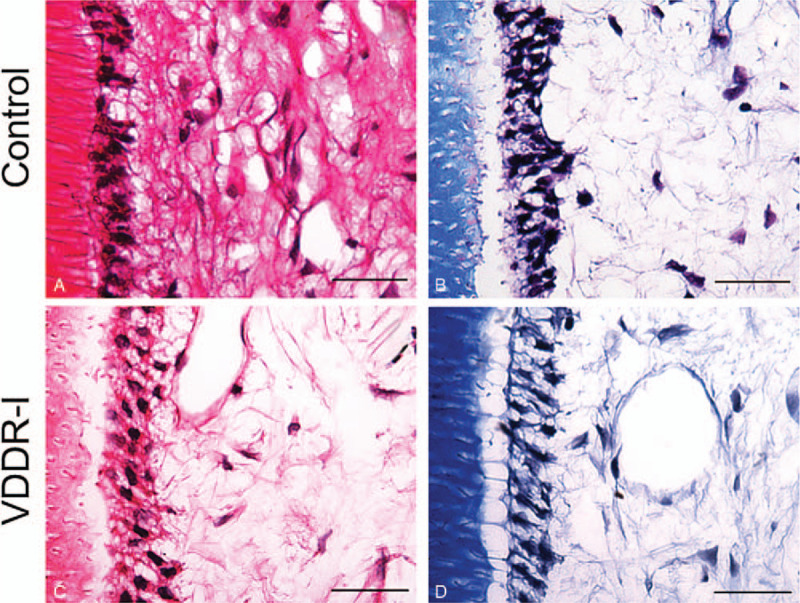
HE and Masson staining. (A, B) HE staining of the control and VDDR-I groups. (C, D) The Masson staining of Control and VDDR-I group. Scale bars: 50 μm.

Moreover, the primary teeth were decalcified with 17% ethylene diamine tetraacetic acid (EDTA) for 2 months and embedded in paraffin. The samples were sliced into 4 μm thick sections and HE and Masson stains were applied to observe the morphological changes of VDDR-I teeth. The odontoblasts from the patient exhibited a sparses arrangement and the layer of odontoblasts showed more vacuolization (Fig. 6).

## 3 Discussion

VDDR-I is a rare type of rickets, which is caused by disturbances in calcium metabolism. This report is the first to describe a long-term dental intervention in a patient with VDDR-I and analyze the changes in tooth morphology.

Rickets is a common worldwide disease caused by disturbances in calcium and phosphate metabolism. Although the symptoms of rickets often include bony deformities and delayed growth, each type of rickets exhibits a variety of clinical manifestations because of different causes.^[3]^ In general, the causes of rickets can be classified into hypocalcemic vitamin D pathway defects, hypophosphatemia, and other defects.^[3,9]^ The hypocalcemic vitamin D pathway defects include nutritional rickets, which are caused by inadequate vitamin D supply,^[10]^ VDDR-I, which is caused by mutations in the *CYP27B1* gene leading to 1-α-hydroxylase deficiency,^[11]^ and vitamin D-dependent rickets type II (VDDR-II), which is caused by mutations in the vitamin D receptor (*VDR*) gene.^[12]^ Regarding hypophosphatemic rickets, X-linked hypophosphatemia (XLH), which is caused by inactivating mutations in the *PHEX* gene,^[13]^ and autosomal dominant hypophosphatemic rickets (ADHR), which is caused by mutations in *FGF23*, dentin matrix protein 1 (*DMP1*) and ectonucleotide pyrophosphatase/phosphodiesterase 1 (*ENPP1*)^[9]^ are the most common forms. Although there are various forms of rickets, the clinical understanding of each form is quite variable. For example, since VDDR-I is a rare autosomal recessive form of rickets, the dental phenotypes of VDDR-I patients are reported much less frequently than the phenotypes of other forms of rickets mentioned above.^[2]^ Studies on VDDR-I focus on sporadic clinical cases and phenotypes of 1-α-hydroxylase-null mice.^[14,15]^ Thus, there is still a lack of evidence regarding long-term dental intervention in VDDR-I. In this case, the type of rickets could be diagnosed by the classical rachitic skeleton with low levels of serum calcium and 1,25-(OH)_2_ vitamin D; and high levels of PTH. The patient had severe oral problems, such as seven decayed teeth, severe enamel defects, and delayed tooth development. With 3 years of dental intervention, the patient exhibited normal tooth replacement, root development, and acceptable oral hygiene status. This indicates that early oral health interventions are effective in maintaining oral health in VDDR-I patients, such as filling early dental caries to prevent further damage, applying metal preformed crowns to protect the first molars, and regular administration of fluoride to promote enamel remineralization and prevent caries.

Considering that calcium and phosphate ions are critical in the process of tooth mineralization, patients suffering from rickets often have additional oral problems, while the oral symptoms are not identical in all types of rickets ^[2]^. For instance, teeth exhibit periapical abscesses in ADHR.^[2]^ Additionally, enamel defects, periodontal disease, and malocclusion occur in XLH.^[5]^ Previously, it was reported that patients with VDDR-I exhibit hypoplastic enamels, large pulp chambers, short roots, and chronic periodontal disease.^[1,14]^ However, there is still a lack of morphologic and histologic evidence regarding VDDR-I. In this report, we detected irregular enamel surface and reduction in both weight percent (Wt%) and atomic percent (At%) of calcium in the patient through SEM and energy dispersive spectrometer examination, confirming the presence of amelogenesis imperfecta. HE and Masson staining showed an abnormal odontoblast arrangement, implying the existence of a dentin defect. However, because the teeth we collected were primary teeth with limited numbers, further research on dentin in VDDR-I patients is needed.

Previous reports have suggested that normal development of the tooth can occur with effective treatment in rickets patients.^[6,16]^ For example, patients with nutritional rickets have the potential to prevent further dental defects by adequate calcium supplementation.^[16]^ Similarly, dental defects in XLH can be prevented by adequate calcium supplementation.^[5]^ This VDDR-I patient, began effective drug intervention at the age of 3 years. After 7 years of calcium supplementation, serum calcium attained normal levels and remission of rickets was observed. However, the newly erupted permanent canine was yellowish and exhibited enamel defects. The findings illustrated that amelogenesis imperfecta could not be prevented by calcium supplementation in VDDR-I patients, suggesting the crucial role of dental intervention.

There has been significantly less research on teeth in patients with rickets than on bone, let alone long-term dental intervention.^[3,17]^ The clinical departments visited by rickets patients are generally pediatrics or endocrinology, not the dental department. Thus, oral abnormalities can often be ignored by endocrinologists or pediatricians. Our findings show that early dental intervention can effectively prevent further dental defects suggesting that multidisciplinary collaborations are important in improving the therapeutic effect of patients with VDDR-I.

## 4 Conclusion

In this report, we describe the clinical features and laboratory findings of VDDR-I, enhancing the understanding of oral symptoms in patients with VDDR-I. Additionally, this report is the first to describe long-term dental intervention in a VDDR-I patient with amelogenesis imperfecta, which was hardly prevented by drug intervention, confirming that long-term dental intervention including metal preformed crown and remineralization therapy can prevent further oral impairment.

## Author contributions

All authors read and approved the final draft of the manuscript.

**Conceptualization:** Kun Xuan, Ke-Wen Wei.

**Data curation:** An-Qi Liu, Li-Shu Zhang, Mei-Ling Wu, Tian-Yi Li.

**Funding acquisition:** Hao Guo, Ke-Wen Wei.

**Methodology:** An-Qi Liu, Li-Shu Zhang, Hao Guo.

**Supervision:** Kun Xuan, Ke-Wen Wei.

**Visualization:** An-Qi Liu, Li-Shu Zhang.

**Writing – original draft:** An-Qi Liu, Li-Shu Zhang.

**Writing – review & editing:** Hao Guo, Mei-Ling Wu, Tian-Yi Li, Kun Xuan, Ke-Wen Wei.

## Abbreviations

Abbreviations: PTH = parathyrin, SEM = scanning electron microscopy, VDDR-I = vitamin D-dependent rickets type I.
